# Supplementary material for: In Vivo Analysis of the Contribution of Proprotein Convertases to the Processing of FGF23
Source: Front Endocrinol (Lausanne). 2021 Jun 4;12:690681. doi: 10.3389/fendo.2021.690681 (PMC8213403; doi:10.3389/fendo.2021.690681)
Supplement: Supplementary Table — Primers used. [file Table_1.docx]

| **Table Appendix: Primers used** | | | | |
| --- | --- | --- | --- | --- |
| **Reagent type (species) or resource** | **Designation** | **Source or reference** | **Identifiers** | **Additional information (sequence)** |
| Sequence-based reagent | Actin beta- For | This paper | QPCR primer (amplify *Actb*, M. musculus) | GACCTCTAT GCCAACACAGT |
| Sequence-based reagent | Actin beta- Rev | This paper | QPCR primer (amplify *Actb*, M. musculus) | AGTACTTGC GCTCAGGAGGA |
| Sequence-based reagent | Slc34a1-Fw | This paper | QPCR primer (amplify *Slc34a1*, M. musculus) | GCATCCTACTGTGGTACCCG |
| Sequence-based reagent | Slc34a1-Rv | This paper | QPCR primer (amplify *Slc34a1*, M. musculus) | CAGCAAACCAGCGGTACTTG |
| Sequence-based reagent | Slc34a3-Fw | This paper | QPCR primer (amplify *Slc34a3*, M. musculus) | CCTTTGGACTTTCCCTGGCA |
| Sequence-based reagent | Slc34a3-Rv | This paper | QPCR primer (amplify *Slc34a3*, M. musculus) | GATGGTCGGTGTTGTTGCAG |
| Sequence-based reagent | Hamp-Fw | This paper | QPCR primer (amplify *Hamp*, M. musculus) | AGAGCTGCAGCCTTTGCAC |
| Sequence-based reagent | Hamp-Rv | This paper | QPCR primer (amplify *Hamp*, M. musculus) | GAGGTCAGGATGTGGCTCTA |
| Sequence-based reagent | Tfrc-Fw | This paper | QPCR primer (amplify *Tfrc*, M. musculus) | TAAATTCCCCGTTGTTGAGG |
| Sequence-based reagent | Tfrc-Rv | This paper | QPCR primer (amplify *Tfrc*, M. musculus) | CAGGACAGCTTCCTTCCATT |
| Sequence-based reagent | Cyp27b1-Fw | This paper | QPCR primer (amplify *Cyp27b1*, M. musculus) | GAAGCTGCGATGAGGAACCA |
| Sequence-based reagent | Cyp27b1-Rv | This paper | QPCR primer (amplify *Cyp27b1*, M. musculus) | TTCCCCACTATGGACTGGACA |
| Sequence-based reagent | Cyp24a1-Fw | This paper | QPCR primer (amplify *Cyp24a1*, M. musculus) | CCCAGCGGCTAGAGATCAAA |
| Sequence-based reagent | Cyp24a1-Rv | This paper | QPCR primer (amplify *Cyp24a1*, M. musculus) | TTTCTTTTGGAAGGCGCTGC |
| Sequence-based reagent | Fgf23-Fw | This paper | QPCR primer (amplify *Fgf23*, M. musculus) | CCGCTGCTGCATTTCTACAC |
| Sequence-based reagent | Fgf23-Rv | This paper | QPCR primer (amplify *Fgf23*, M. musculus) | CTTGAGCACGTTCAGTGGGT |
| Sequence-based reagent | Epor-Fw | This paper | QPCR primer (amplify *Epor*, M. musculus) | GGACACAAAGGGTGGAGGTC |
| Sequence-based reagent | Epor-Rv | This paper | QPCR primer (amplify *Epor*, M. musculus) | GCTCGAACAGCGAAGGTGTA |
| Sequence-based reagent | Erfe-Fw | This paper | QPCR primer (amplify *Erfe*, M. musculus) | GTTCCAGCTGTTGCTGAAAGG |
| Sequence-based reagent | Erfe-Rv | This paper | QPCR primer (amplify *Erfe*, M. musculus) | TACCCGAGGCTGGTGTAGTG |
